# Genetic characterization of canine astrovirus in non-diarrhea dogs and diarrhea dogs in Vietnam and Thailand reveals the presence of a unique lineage

**DOI:** 10.3389/fvets.2023.1278417

**Published:** 2023-09-25

**Authors:** Tin Van Nguyen, Chutchai Piewbang, Somporn Techangamsuwan

**Affiliations:** ^1^The International Graduate Program of Veterinary Science and Technology (VST), Faculty of Veterinary Science, Chulalongkorn University, Bangkok, Thailand; ^2^Faculty of Animal Science and Veterinary Medicine, Nong Lam University, Ho Chi Minh City, Vietnam; ^3^Animal Virome and Diagnostic Development Research Unit, Faculty of Veterinary Science, Chulalongkorn University, Bangkok, Thailand; ^4^Department of Pathology, Faculty of Veterinary Science, Chulalongkorn University, Bangkok, Thailand

**Keywords:** canine astrovirus, ORF2, phylogenetic tree, Vietnam, Thailand

## Abstract

The role of canine astrovirus (CaAstV) in canine gastrointestinal disease (GID) is unknown. In this study, a total of 327 fecal swab (FS) samples were collected, including 113 FSs in Vietnam (46 samples from healthy dogs and 67 samples from GID dogs) and 214 FSs in Thailand (107 samples from healthy dogs and 107 samples from GID dogs). Overall, the prevalence of CaAstV in Vietnam and Thailand was 25.7% (29/113) and 8.9% (19/214), respectively. CaAstV was detected in both non-diarrhea dogs (21.7 and 7.5%) and diarrhea dogs (28.4% and 10.3%), respectively, in Vietnam and Thailand. In both countries, CaAstV was frequently detected in puppies under 6 months of age (23.3%) (*p* = 0.02). CaAstV-positive samples in Vietnam and Thailand were identified as co-infected with canine parvovirus, canine enteric coronavirus, canine distemper virus, and canine kobuvirus. The complete coding sequence of seven Vietnamese CaAstV and two Thai CaAstV strains were successfully characterized. Phylogenetic analyses showed that Vietnamese and Thai CaAstV strains were genetically close to each other and related to the Chinese strains. Furthermore, analysis of complete coding sequences indicated that the OR220030_G21/Thailand/2021 strain formed a unique lineage, whereas no recombination event was found in this study, suggesting that this strain might be an original lineage. In summary, this is the first study to report the presence of CaAstV in dogs with and without diarrhea in Vietnam and Thailand, and it was most often found in puppies with diarrhea. Our results highlight the importance of the CaAstV in dog populations and the need for continued surveillance of these emerging pathogens.

## 1. Introduction

Canine astrovirus (CaAstV) is a star-shaped, non-enveloped, single-stranded, positive-sense RNA virus with approximately 30-nm in size (1). The CaAstV belongs to the genus *Mamastrovirus*, the family *Astroviridae*. Currently, the *Mamastrovirus* genus includes 19 viral species (*Mamastrovirus* 1–19), and CaAstV belongs to the *Mamastrovirus 5*.^1^ Although CaAstV has been discovered in the feces of diarrhea dogs by electron microscopy since 1980, the entire genome structure of CaAstV was not reported until 2015 (2). The viral genome, with a length of 6.6 kilobases (kb), contains three main open reading frames (ORFs). The main ORFs include ORF1a and ORF1b, which are present at the 5′ end and encode a nonstructural protein, including the viral protease and RNA-dependent RNA polymerase. The ORF2, presenting at the 3′ end, encodes a capsid protein (3). Based on an analysis of the capsid protein of CaAstV, ORF2 is considered the most antigenic determinant region among different CaAstV strains (4). The hypervariable region of the capsid protein of CaAstV, therefore, may play an important role in virus attachment to target cells by forming virion spikes and interacting with cellular receptors (5, 6); in viral neutralization through the binding of neutralizing monoclonal antibodies with these variable regions; and in heterologous immunity (5, 7).

The host range of astroviruses is comprised of many mammalian species, such as cows (8), buffalo calves (9, 10), sheep (11), pigs (12), cats (13), mink (14), bats (15), red deer (16), urban brown rats (17), synanthropic squamates (18), California sea lions, Steller’s sea lions, and bottlenose dolphins (19), as well as certain avian species (chicken, turkey, duck) (20, 21). Infected hosts present the main clinical features of gastrointestinal diseases (GIDs). However, astrovirus infection was also reported to be associated with extra-intestinal diseases, such as hepatitis and nephritis, in various species, including duck (22, 23), goose (24), and mink (25, 26). In humans, besides norovirus and rotavirus infections, astrovirus is considered to be an 10% of etiological agents associated with gastroenteritis in children globally (27, 28). Furthermore, astrovirus can cause severe disseminated infection and death in highly immunocompromised pediatric patients (29).

Regarding CaAstV infection in domestic dogs, GIDs have been commonly observed and reported in various studies in China, Japan, Italy, the United Kingdom, and Australia. These findings suggest that there is a positive correlation between CaAstV infection and GID in dogs (3, 4, 30–32). In wild carnivores, CaAstV was coinfected with canine distemper virus (CDV) in crab-eating foxes (*Cerdocyon thous*) showing neurological signs (33). In addition, coinfection of CaAstV with other enteric viruses, such as canine parvovirus (CPV) and canine coronavirus (CCoV), has also been frequently reported (3, 34). Conversely, other studies have reported a negative association between the presence of CaAstV and gastroenteritis (35, 36). Therefore, the pathogenesis and clinical significance of CaAstV infection in dogs remains uncertain (37).

Many investigations have shown that the genetic structure of CaAstV changes over time through genetic recombination. Virus recombination is one of the events that plays a key role in virus evolution and infectivity, including the expansion of the viral host range, increased virulogenicity, evasion of host immunity, and antiviral resistance (38). Genetic recombination among CaAstV strains has been reported in recent years (39, 40), indicating that it is a potential process for CaAstV evolution. Recently, recombination of the astrovirus has been associated with zoonotic potential by illustrating the genetic recombination between human astrovirus (HAstV) and porcine astrovirus (PoAstV) (41), as well as the emergence of a new strain of astrovirus found in fecal samples obtained from children with diarrhea in Nigeria, presented genetically close to CaAstV (42). These findings suggest a potential risk for the emergence of a novel astrovirus in humans that initially emerged from animal origins. Information regarding the genetic characterization and recombination analysis of CaAstV in countries that are not currently being investigated is necessary. Therefore, this study aimed to investigate the presence of CaAstV in feces obtained from dogs in Vietnam and Thailand that have not previously been reported, and to compare the genomic characterization of CaAstV between Vietnamese and Thai strains and with other available strains deposited in the database. The clinical significance and the association of CaAstV-positive dogs with the risk factors of positive dogs were also evaluated.

## 2. Materials and methods

### 2.1. Animals and sample collection

Fecal swab (FS) samples were randomly collected from apparently healthy dogs and dogs clinically presenting with GIDs (watery diarrhea, bloody diarrhea) from veterinary hospitals in Vietnam (Ho Chi Minh City) and Thailand (Bangkok and Nakhon Si Thammarat Province) from August 2021 to August 2022. A total of 327 FSs included 113 FSs in Vietnam (46 samples from healthy dogs and 67 samples from GID dogs) and 214 FSs in Thailand (107 samples from healthy dogs and 107 samples from GID dogs). A questionnaire was conducted to collect information on age, breed, sex, and vaccination status. The inclusion criteria for fecal sampling were dogs presenting with clinical symptoms of bloody diarrhea or watery diarrhea when visiting hospitals. While dogs were showing GI signs from food poisoning, liver and biliary disease, or dogs were vaccinating 4 weeks prior, samplings were excluded from this study. All studied dogs were divided into 6 different age groups, modified according to a categorization of the domestic dog age groups (43) for further interpretation on risk factor analysis (Table 1).

**Table 1:** Prevalence of canine astrovirus (CaAstV) in Vietnam and Thailand.

Age groups (month)	Number of CaAstV-positive dogs/Total collected dogs (%)								
	Vietnam			Thailand			Overall		
	Non-diarrhea	Diarrhea	Total	Non-diarrhea	Diarrhea	Total	Non-diarrhea	Diarrhea	Total
Puppies (≤ 6)	3/15 (20%)	15/46 (32.6%)	18/61 (29.5%)	5/38 (13.2%)	5/21 (23.8%)	10/59 (16.9%)	8/53 (15.1%)	20/67 (29.9%)	28/120 (23.3%)
Juveniles (> 6–12)	3/15 (20%)	2/9 (22.2%)	5/24 (20.8%)	0/8 (0%)	2/17 (11.8%)	2/25 (8%)	3/23 (13%)	4/26 (15.4%)	7/49 (14.3%)
Young adults (> 12–24)	2/3 (66.7%)	1/3 (33.3%)	3/6 (50%)	1/9 (11.1%)	0/11 (0%)	1/20 (5%)	3/12 (25%)	1/14 (7.1%)	4/26 (15.4%)
Mature adults (> 24–72)	2/12 (16.7%)	1/9 (11.1%)	3/21 (14.3%)	1/21 (4.8%)	2/23 (8.7%)	3/44 (6.8%)	3/33 (9.1%)	3/32 (9.1%)	6/65 (9.2%)
Senior (> 72–132)	0/1 (0%)	NA	0/1 (0%)	1/18 (5.6%)	2/25 (8%)	3/43 (7%)	1/19 (5.3%)	2/25 (8%)	3/44 (6.8%)
Geriatric (> 132)	NA	NA	NA	0/13 (0%)	0/10 (0%)	0/23 (0%)	0/13 (0%)	0/10 (0%)	0/23 (0%)
**Total**	**10/46 (21.7%)**	**19/67 (28.4%)**	**29/113 (25.7%)**	**8/107 (7.5%)**	**11/107 (10.3%)**	**19/214 (8.9%)**	**18/153 (11.8%)**	**30/174 (17.2%)**	**48/327 (14.7%)**

NA, not available sample.

The FS sampling procedure was performed using a sterile disposable cotton swab (Puritan, Guilford, United States). The swab was inserted into the rectum, then subsequently immersed in 1 mL of 1% sterile phosphate buffered brine (PBS) and kept at −80°C until the extraction step. The animal study was reviewed and approved by Institutional Animal Care and Use Committee (IACUC) (No. 2231006) and the Institutional Biosafety Committee (IBC) (No. 2131019) of Chulalongkorn University (Bangkok, Thailand), and approval from Animal Ethics Committee (AEC) (No. NLU-220217) of Nong Lam University (Ho Chi Minh City, Vietnam).

### 2.2. RNA extraction

Viral genomic RNA extraction was performed using the Viral Nucleic Acid Extraction Kit II (Geneaid, Ltd., Taipei, Taiwan) following the manufacturer’s protocol. Total extracted RNA quantity and quality were measured using a Nanodrop^®^ Lite spectrophotometer (Thermo Fisher Scientific Inc., Waltham, MA, United States) at absorbance ratio A260/A280 (44). The extracted samples were kept at −80°C until used.

### 2.3. CaAstV detection

The extracted RNA samples were subjected to CaAstV detection using reverse-transcription PCR (RT-PCR). Briefly, a total final mixture volume of 25 μL for QIAGEN^®^ One-step RT-PCR Kit (Qiagen GmbH, Hilden, Germany), comprising 5 μL of 5x QIAGEN buffer, 1 μL of 10 mM dNTP Mix, 2 μL of 0.6 μM forward and reverse primers targeting the ORF1b region of the CaAstV (Supplementary Table S1), 1 μL of Enzyme mix, 3 μL of extracted RNA, and distilled water to make up 25 μL final volume, was performed on a thermocycler (SensoQuest GmbH, Göttingen, Germany). The thermocycling conditions consisted of a complementary DNA (cDNA) synthesis step at 50°C for 30 min, and subsequently proceeded to an initial PCR activation step at 95°C for 15 min, following 40 cycles of denaturation at 95°C for 30 s, annealing at 53°C for 30 s and extension at 72°C for 1 min, and a final extension step at 72°C for 10 min. The positive control for CaAstV was synthesized by GeneArt™ Strings™ DNA Fragments based on the ORF1b region of strain MN882002 (Thermo Fisher Scientific GmbH, Darmstadt, Germany). A no-template control (NTC) was used as the negative control. The PCR products were visualized by using QIAxcel^®^ DNA Screening Kit (Qiagen GmbH, Hilden, Germany) and the Qiaxcel^®^ high-resolution capillary electrophoresis instrument (Qiagen GmbH, Hilden, Germany). The settings and analysis methods for DNA fragments were set as previously reported (45). The presence of a 290-bp amplicon was considered positive. To confirm the presence of CaAstV nucleotide sequences, the PCR products were submitted for genetic sequencing using the next-generation (NGS)-based method (Celemics, Inc., Seoul, South Korea). The derived nucleotide sequences were analyzed and compared to previously described CaAstV deposited in the GenBank database using BLASTn analysis.

To determine the presence of other concomitant viruses, the CaAstV-positive samples were subsequently screened for common canine enteric viruses, including CPV (46), CDV (47), CCoV (48), and canine kobuvirus (CaKoV) (49).

### 2.4. CaAstV whole genome characterization

The positive samples from the CaAstV-PCR screening were further investigated to complete the full-length genome analysis using multiple PCR assays. Sets of primer pairs used for full-length genome amplification of CaAstV were designed based on nucleotide alignments of previously described CaAstV sequences available in the GenBank database (Supplementary Table S1). First, cDNA was constructed from the extracted RNA samples using Omniscript^®^ Reverse-transcription Kit (Qiagen GmbH, Hilden, Germany). A final volume of 20 μL for cDNA construction was comprised of 2 μL of 10x buffer RT, 2 μL of 5 mM dNTP Mix, 1 μL of 10 μM Random primer (Promega, Madison, Wisconsin, United States), 1 μL of RNase-free water, 0.75 μL of 1x buffer, 0.25 μL of RNase inhibitor (10 units/μL), 1 μL of Omniscript Reverse Transcriptase, and 12 μL of extracted RNA sample. After that, PCR proceeded in a final volume of 25 μL containing 3 μL of cDNA, 1 μL of 1 μM of each forward and reverse primer, 12.5 μL GoTaq^®^ Green Master Mix (Promega, Madison, Wisconsin, United States), and distilled water. The thermocycling condition consisted of an initial denaturation at 95°C for 5 min, followed by 40 cycles at 95°C for 1 min, 51–55°C for 1 min, and 72°C for 1 min, and then a final extension at 72°C for 10 min. The PCR products were visualized by QIAxcel^®^ DNA Screening Kit, as mentioned above.

The positive amplicons were subjected to genetic sequencing using the protocol described above. Subsequently, the derived genetic sequences were aligned and assembled using the BioEdit software package version 7.2 with the ClustalW function.

### 2.5. Phylogenetic and genetic analyses

Genetic analysis was performed by comparing the homology of the nucleotide sequences of CaAstV obtained from this study with those of CaAstV available in the database. The phylogenetic tree based on the whole genome, ORF1a, ORF1b, and ORF2 regions of CaAstV was constructed using the MEGA software package version 10.0. The maximum likelihood (ML) method, based on the General Time Reversible model (GTR) (for whole genome, ORF1a, and ORF2), and the Tamura 3-parameter model (T92) (for ORF1b), with a gamma distribution and invariable sites (G + I), together with 1,000 bootstrap replicates, was used to evaluate the relationship between these obtained CaAstV strains and the other strains. The nucleotide and deduced amino acid sequences of the CaAstVs were aligned and compared using the BioEdit software package, version 7.2.

### 2.6. Recombination analysis

Genetic recombination events of all obtained CaAstV strains in Vietnam and Thailand were screened using the Recombinant Detection Program software package version 4.0 (RDP4). Seven integrated recombinant detection algorithms, including RDP, GeneConv, Chimera, MaxChi, SiScan, 3Seq, and BootScan, were used to identify genetic recombination. The potential recombination sequences were considered when there were positives in at least 4 out of 7 methods with *p*-values ≤0.01. These sequences were subsequently subjected to further analysis using a similarity plot and Bootscan analysis embedded in SimPlot v. Beta 4.94 software package to illustrate recombination breakpoints. The analysis followed a previous publication (45).

### 2.7. Statistical analysis

The associations between the presence of CaAstV and variables, including the clinical presentation of sampled dogs (non-diarrhea and diarrhea dogs) and age group, were analyzed using Pearson’s chi-squared test or Fisher’s exact test (depending on the population size for each variable). The relationship was considered statistically significant when the *p*-value was <0.05. In addition, the odds ratio (OR) was calculated to quantify the strength of the association between each factor and the presence of CaAstV. Statistical analyses were performed using SAS^®^ Studio software (© 2022 SAS Institute Inc., Cary, NC, United States).

## 3. Results

### 3.1. Prevalence of CaAstV infection in domestic dogs in Vietnam and Thailand

Overall, the prevalence of CaAstV in Vietnam and Thailand was 25.7% (29/113) and 8.9% (19/214), respectively. The prevalence of CaAstV detection in Vietnam was higher than in Thailand, with a significant difference (*p* = 0.0001; OR = 3.5; 95% CI: 1.8823–6.6697). Based on the clinical manifestations of the two survey groups, CaAstV was detected in rectal swab samples obtained from non-diarrhea dogs (21.7% and 7.5%) and diarrhea dogs (28.4% and 10.3%), respectively, in Vietnam and Thailand. The pooled CaAstV prevalence of the two countries between non-diarrhea and diarrhea dogs was 11.8% (18/153) and 17.2% (30/174), respectively. However, there was no statistical difference in the prevalence of CaAstV infection between the two dog groups (*p* = 0.16). CaAstV was detected in dogs of various ages (Table 1). For overall detection in both countries, the highest CaAstV prevalence (23.3%) was in puppies, and it was higher than in mature adults (*p* = 0.02; OR = 2.99; 95% CI: 1.1686–7.6644) and seniors, with a significant difference (*p* = 0.02; OR = 4.16; 95% CI: 1.1961–14.4637).

For CaAstV-positive samples found in Vietnam, 31% (9/29), 20.7% (6/29), and 44.8% (13/29) were co-detected with individual CPV, CCoV, and CaKoV, respectively, whereas 3.4% (1/29) of CaAstV-positive cases were co-detected with CPV, CCoV, and CaKoV, and another 3.4% (1/29) were co-detected with CPV, CCoV, CaKoV, and CDV. In Thailand, 5.3% (1/19) and 31.6% (6/19) of CaAstV-positive dogs were co-detected with only CCoV and CaKoV, respectively. None of the samples was co-detected with CPV, CDV, or other triple infections found in Thailand.

### 3.2. Genetic characterization of the CaAstV in Vietnam and Thailand

In this study, 7 and 2 complete coding sequences of CaAstV strains [excluding poly (A) tails, 3′UTRs, and 5′UTRs] obtained from Vietnam and Thailand were, respectively, characterized. Mostly, the Vietnamese and Thai CaAstVs contained 6,465 nucleotide (nt) lengths, including ORF1a (2,670 nt), ORF1b (1,536 nt), and ORF2 (2,496 nt). Among these, the CaAstV strain OR220030_G21/Thailand/2021 contained 6,447 nt lengths, including ORF1a (2,670 nt), ORF1b (1,536 nt), and ORF2 (2,478 nt). These CaAstV nucleotide sequences were submitted to the GenBank database under accession numbers OR220022-OR220030. Details regarding the samples used for the complete coding sequences are indicated in Table 2.

**Table 2:** Detail information of Thai and Vietnamese canine astrovirus (CaAstV) strains obtained from this study.

Dog no.	Time of collection	City, country^a^	Sex^b^	Age (month)	Breed	Clinical signs	Housing	Vaccination history	Deworming	Interact with other pets	GenBank accession number
V91	May 2022	HCM, VN	M	2	Vietnam Local	Diarrhea	Indoor, outdoor	No	No	Dogs, cats	OR220022
V194	Apr 2022	HCM, VN	M	2	Mixed	Diarrhea	Indoor, outdoor	Yes	Yes	Dogs	OR220023
V196	Apr 2022	HCM, VN	M	2	Mixed	Diarrhea	Indoor, outdoor	No	No	Dogs, cats	OR220024
V138	May 2022	HCM, VN	M	3	Dachshund	Diarrhea	Indoor	Yes	No	No	OR220025
V98	Apr 2022	HCM, VN	M	3	Mixed	Diarrhea	Indoor, outdoor	No	No	No	OR220026
V107	Apr 2022	HCM, VN	F	3	Mixed	Diarrhea	Indoor, outdoor	No	No	No	OR220027
V111	May 2022	HCM, VN	F	7	Mixed	Healthy	Indoor, outdoor	No	No	No	OR220028
S76	Sep 2021	BKK, TH	M	3	Yorkshire	Healthy	Indoor, outdoor	Yes	Yes	Dogs	OR220029
G21	Dec 2021	BKK, TH	F	2	Mixed	Diarrhea	Outdoor	No	No	Dogs	OR220030

a BKK, TH (Bangkok, Thailand); HCM, VN (Ho Chi Minh city, Vietnam).

b M (Male); F (Female).

The nucleotide and amino acid similarities of Vietnamese and Thai CaAstVs were compared with other strains found in China, Hungary, England, Australia, India, and the United States that were previously deposited in the GenBank database. Among the seven complete coding sequences of Vietnamese CaAstVs, they shared 96.3%–97.8% nt and 94.2%–97.7% aa similarity to each other. Between the two complete coding sequences of Thai CaAstVs, they had 91.1% nt and 82.4% aa similarities. When compared between the CaAstVs complete coding sequences obtained from Vietnam and Thailand, they shared 90.5%–96.3% nt and 83.1%–94.8% aa similarities, and they also had the highest nt similarity to China strains (MN882007.1/2018 and MN882009.1/2019) (Supplementary Table S2).

As expected, the results showed that ORF2 was the most variable region for the CaAstV, ranging from 73% to 99% and 70.3% to 98.5% for nt and aa similarities, respectively (Supplementary Table S2). Analysis of the nt and aa sequences within ORF2 revealed differences in size between strains. Most of the CaAstV strains obtained from this study had a length of 2,496 nt encoding 831 aa, except for the strain OR220030_G21/Thailand/2021 that had 2,478 nt length and encoded 825 aa and resulted in the 18-nt (6-aa) shorter ORF2 of the OR220030_G21/Thailand/2021 than others. In addition, the ORF2 gene regions of the obtained Vietnamese and Thai CaAstVs were compared with other available strains from the database to assess aa variations (Supplementary Table S3). The result showed that motif insertion of 7 consecutive aa (PTIEEEQ) (position 733–739) was evident in almost all Vietnamese and Thai CaAstV strains but excepted for CaAstV strain OR220030_G21/Thailand/2021 that had an exclusive insertion of 1 aa (Serine) at position 669 (Supplementary Table S3). Additionally, CaAstV strain OR220030_G21/Thailand/2021 ORF2 region exhibited low sequence identity to a OR220029_S76/Thailand/2021 strain and other reference strains available in GenBank database (Supplementary Table S2). Notably, no recombination events were found within the CaAstV strains obtained in this study.

### 3.3. Phylogeny of the Vietnam and Thailand CaAstV

**Figure 1:**
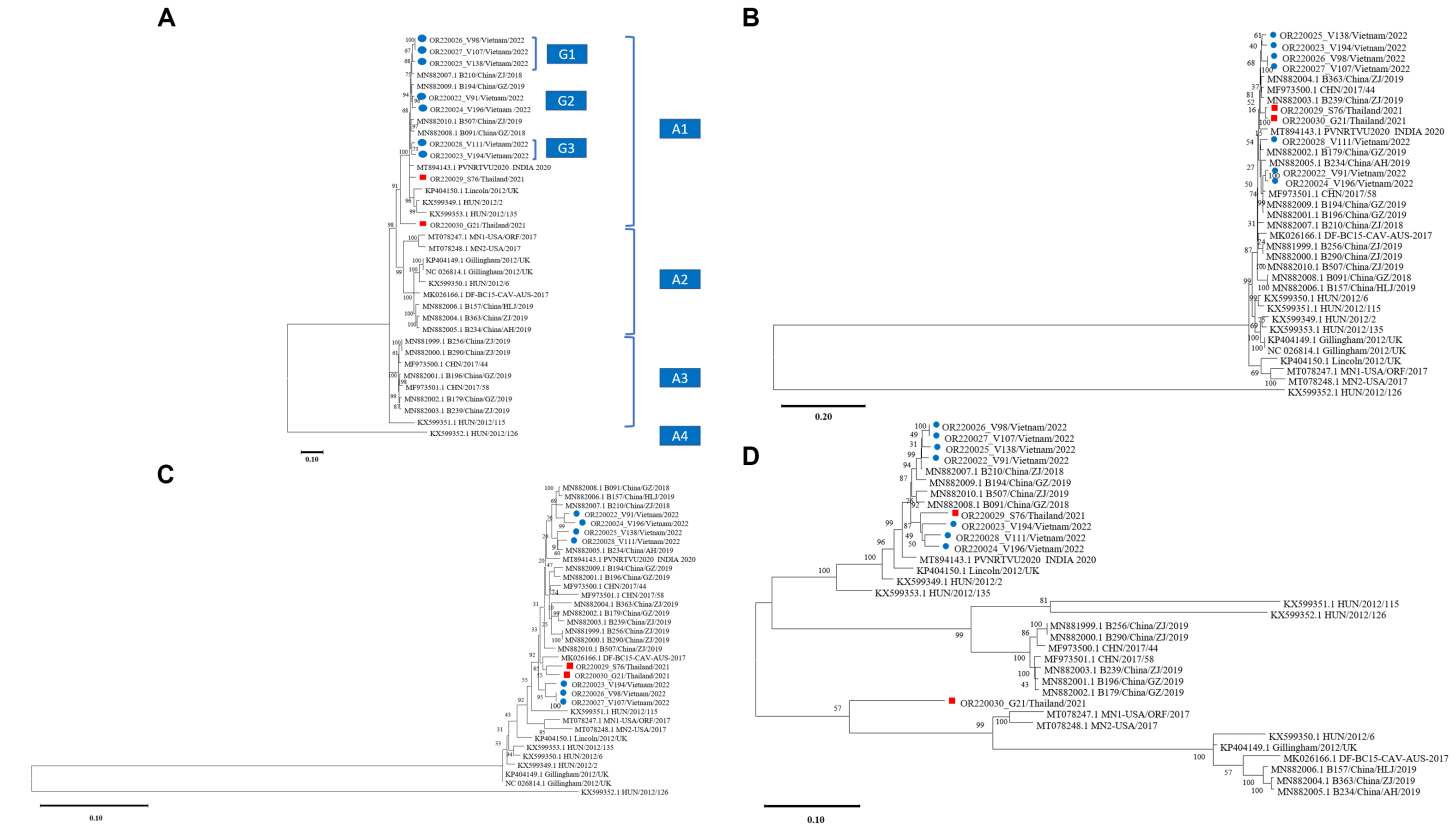
Phylogenetic trees of canine astrovirus (CaAstV): **(A)** complete coding sequences, **(B)** ORF1a region, **(C)** ORF1b region, and **(D)** ORF2 region. Trees of complete coding sequences and ORF1a were constructed using MEGA v10.0 with the neighbor-joining algorithm with the general time-reversible model, while trees of ORF1b and ORF2 were constructed with the neighbor-joining algorithm with the Tamura-3-parameter model and the general time-reversible model, respectively. All trees were run with 1,000 replications of bootstrap analysis. ● indicates Vietnamese CaAstV isolates and ■ indicates Thai CaAstV isolates in this study.

The phylogenetic tree based on the nucleotide sequences of the nine complete coding sequences of the CaAstV, ORF1a, ORF1b, and ORF2 strains of Vietnam and Thailand are shown in Figure 1. Overall, the phylogenic topologies based on complete coding sequences and the studied genes were similarly presented with some discrepancy details. The complete coding sequence-based phylogenetic tree showed that CaAstV strains were divided into 4 major subgroups: A1, A2, A3, and A4. The CaAstV strains found in Vietnam and Thailand were clustered together and located in Cluster A1. Within the A1 subgroup, CaAstV Vietnamese strains were separated into 3 different clusters (G1–G3) and shared a genetic relationship with the Chinese CaAstV strains (Figure 1A). Interestingly, the two CaAstV strains found in Thailand were separated into 2 new lineages. The CaAstV OR220030_G21/Thailand/2021 strain shared a genetic origin, with strains originating in Europe, China, Vietnam, and India. A phylogenetic tree based on the ORF1a and ORF1b regions revealed that Vietnamese and Thai CaAstVs were grouped together as a single clade within the Chinese CaAstVs, which were separated from the European and US strains (Figures 1B,C).

Based on the analysis of the ORF2 region, all strains from this study were divided into two groups. Vietnamese and a OR220029_S76/Thailand/2021 CaAstV strains were clustered together and shared a genetic relationship with the Chinese CaAstV strains. However, the CaAstV OR220030_G21/Thailand/2021 strain was distinct by creating a single clade (Figure 1D).

## 4. Discussion

CaAstV has been detected in many countries, including China, the United Kingdom, Italy, France, the United States, Korea, and Brazil; however, no study has been conducted, not only in Thailand and Vietnam, but also in other countries in Southeast Asia. In this study, we found that CaAstV was prevalent in Vietnam and Thailand. Previous studies on CaAstV have only focused on puppies, except for studies in the United Kingdom (3) and Japan (31) that investigated the presence of CaAstV in dogs of all ages. Therefore, when comparing the prevalence between countries, there are some discrepancies due to the different age groups of the studied animals, which may result from different geography. In this study, in either Vietnam or Thailand, almost all the dogs investigated were predisposed to CaAstV infection, especially in puppies. Furthermore, many studies have shown a correlation between infection in infancy and the clinical severity of astrovirus infection, not only in puppies but also in humans and other species (4, 14, 28, 30, 35). Indeed, antibodies specific to CaAstV tend to form in dogs over 3 months of age, and conversely, almost dogs under 3 months of age do not have this antibody specificity, making puppies susceptible to CaAstV infection (4). Similar to our investigation that found CaAstV in older dogs, there are previous reports of AstV detection in adult dogs (3) and humans who are elderly and immunocompromised (50).

In this study, CaAstV was detected in FSs obtained from dogs with and without diarrhea. This result was consistent with previous studies conducted in various countries, including Italy (4), Hungary (36) and China (40, 51). However, our findings contrast to the other studied reports in China (30), England (3), Japan (31), and Brazil (33) where the CaAstV was only present in diarrhea dogs. In agreement with the results of this study, a study revealing a negative association between the presence of astrovirus and abnormal feces in puppies was also noted (35). Astrovirus-containing genetic mutation has been proposed to be associated with viral adaptation, leading to greater resistance to an extreme environment (52), and probably resulting in persistent infection. However, we could not find a significant genetic mutation associated with or without diarrhea in the CaAstV sequences obtained from this study. Thus, further experimental and clinical observations regarding CaAstV pathogenic strains are needed, as previously speculated in the results of the PoAstV study (53).

The presence of CaAstV in GID dogs has been reported as a coinfection with other major enteric viral pathogens (4, 39, 51, 54); however, a single infection of CaAstV also results in enteric disease (4). These findings may indicate that CaAstV may be involved in diarrhea, either as a primary or co-secondary pathogen. Since the exact role of CaAstV remains unknown, further observational studies or animal experiments are needed to better understand the pathogenic role of CaAstV. There are studies attempting *in vitro* isolation of CaAstV; the results have been either successful isolation (4, 55) or unsuccessful isolation (3, 39). Therefore, the cultivation of CaAstV remains a major challenge at present.

For the phylogenetic tree based on the complete coding sequences, it was found that the CaAstV Vietnamese and Thai strains were separated into different subclusters, and the CaAstV strain OR220030_G21/Thailand/2021 presented a unique lineage. Phylogenetic analysis of the three regions of CaAstV showed that almost all strains of Vietnam and Thailand tended to cluster together, except for the ORF2 region of the two Thai strains, which were separated by forming a single group. This difference may be due to independent evolution and/or evolutionary constraints for different genomic regions of CaAstV under different selection pressures (56). Indeed, further genomic analysis for the hypervariable region of ORF2, all Vietnamese CaAstVs, and OR220029_S76/Thailand/2021 strains illustrated the motif insertion of 7 consecutive amino acids (PTIEEEQ). The same motif has also been reported in China’s origin strains (39, 40). However, strain OR220030_G21/Thailand/2021 did not have the same mutated motif as found in Vietnamese and Thai strains but had an exclusive 1 aa insertion mutation. In general, it seems that the capsid properties of CaAstV are similar to those of HAstV, in which deletion, insertion, and substitution mutations frequently occur and may affect certain viral functions (30). Indeed, small changes in capsid sequence and structure in HAstV strains can also lead to changes in the virus’s ability to bind, enter, and uncoat (57). However, the motif-mutated 7 consecutive amino acids in this study were all located in the acidic region of the ORF2 region outside the caspase cleavage site to truncate the full-length capsid protein (VP90) to the mature form (VP70) (58, 59). Therefore, the actual role of these mutations in CaAstV structure and function needs to be further investigated.

Besides the exclusive difference in mutations in the capsid region compared with Vietnamese and Thai CaAstV strains, the capsid sequence of strain OR220030_G21/Thailand/2021 showed genetic heterogeneity. Furthermore, genetic recombination was not detected for the Vietnam and Thailand CaAstV strains in this study. The obtained results, together with the significant findings, may suggest that the OR220030_G21/Thailand/2021 strain may be original or may serve a regional character. However, only 2 Thai CaAstV complete coding sequences were identified in this study. Therefore, increasing the number of Thai CaAstV complete coding sequences will elaborate on the significance.

## 5. Conclusion

CaAstV was detected, both in non-diarrhea and diarrhea dogs, at almost ages, with the highest prevalence in dogs less than 6 months old. In addition, CaAstV was found as an individual infection or coinfection with other canine GI viruses (CPV, CDV, CCoV, and CaKoV). Phylogenetic analysis and genomic characterization showed that the CaAstV Vietnamese and Thai strains were closely related to each other and to the Chinese strains. Furthermore, the CaAstV Thai strains were unique. As this study is the first report on CaAstV in Vietnam and Thailand, it is necessary to expand the survey area to better understand the epidemiology and evolution of CaAstV.

## Data availability statement

The original contributions presented in the study are publicly available. This data can be found here: https://www.ncbi.nlm.nih.gov/genbank/, OR220022-OR220030.

## Ethics statement

The animal studies were approved by the Institutional Animal Care and Use Committee (IACUC) (No. 2231006) of Chulalongkorn University (Bangkok, Thailand), The Institutional Biosafety Committee (IBC) (No. 2131019) of Chulalongkorn University (Bangkok, Thailand), and Animal Ethics Committee (AEC) (No. NLU-220217) of Nong Lam University (Ho Chi Minh City, Vietnam). The studies were conducted in accordance with the local legislation and institutional requirements. Written informed consent was obtained from the owners for the participation of their animals in this study.

## Author contributions

TN: Methodology, Data curation, Formal analysis, Investigation, Software, Writing–original draft. CP: Methodology, Conceptualization, Supervision, Writing–review & editing. ST: Conceptualization, Funding acquisition, Methodology, Supervision, Writing–review & editing.

## Footnotes

^1^ https://talk.ictvonline.org/taxonomy/
