# Supplementary material for: Genetic characterization of canine astrovirus in non-diarrhea dogs and diarrhea dogs in Vietnam and Thailand reveals the presence of a unique lineage
Source: Front Vet Sci. 2023 Sep 25;10:1278417. doi: 10.3389/fvets.2023.1278417 (PMC10561284; doi:10.3389/fvets.2023.1278417)
Supplement: Supplementary file 1 [file Table_1.DOCX]

Supplementary Material

**Genetic characterization of canine astrovirus in non-diarrhea dogs and diarrhea dogs in Vietnam and Thailand reveals the presence of a unique lineage**

**Tin V. Nguyen,^1,2^ Chutchai Piewbang,^2,3,^* and Somporn Techangamsuwan^2,3,^***

^1^The International Graduate Program of Veterinary Science and Technology (VST), Faculty of Veterinary Science, Chulalongkorn University, Bangkok 10330, Thailand.

^2^Animal Virome and Diagnostic Development Research Unit, Faculty of Veterinary Science, Chulalongkorn University, Bangkok 10330, Thailand.

^3^Department of Pathology, Faculty of Veterinary Science, Chulalongkorn University, Bangkok 10330, Thailand.

**Table S1** List of primers used for detection^a^ and amplification^b^ the 6 regions of CaAstV genome

| **Direction** | **Sequence (5’-3’)** | **Genome position** | **Product size (bp)** |
| --- | --- | --- | --- |
| **Primers to detect CaAstV** | | | |
| Forward | GYACTATACCRTCTGATTTRATT | 3424 – 3441 | 292 |
| Reverse | ACCARGGTGTCATAGTTCA | 3697 – 3715 |  |
| **Primers to amplify the 6 regions of CaAstV genome** | | | |
| Forward | TCGCCCATAAGATCTCTAGT | 41 – 60 | 1303 |
| Reverse | CCAGTTCTRACCTGCTTCA | 1343 – 1325 |  |
| Forward | TGTGTTGTDTGCCTTTCTTT | 1051 – 1070 | 1433 |
| Reverse | ATGTTYTTTGCATCCTCAGC | 2483 – 2464 |  |
| Forward | GACCCAGAGCTAAGTGATGA | 2218 – 2237 | 1332 |
| Reverse | TTCGGCATTAACCAAACTCC | 3549 – 3530 |  |
| Forward | TATGTTAGGATTGGYGCGGTA | 3304 – 3324 | 1167 |
| Reverse | AGTAGATGTAGCAGTYTGGGT | 4471 – 4451 |  |
| Forward | GACGAAACAGACCTAACAATC | 4353 – 4373 | 1078 |
| Reverse | GGTGMATTCATCTGMACAAA | 5430 – 5411 |  |
| Forward | TYAAAGGYGGYTGGTGGT | 5247 – 5264 | 1343 |
| Reverse | AACCTGTACCCTCGATCCTA | 6589 – 6570 |  |

^a^: Primers were designed based on the available canine astrovirus sequence MN882002.

^b^: Primers were designed based on the available canine astrovirus sequence KX599349.

**Table S3** Comparison of amino acid changes in the ORF2 region of Vietnamese and Thai CaAstVs

| **Accession no.** | **Strain** | **Year** | **Country** | **Amino acid position** | | | | | | | | | | | | |
| --- | --- | --- | --- | --- | --- | --- | --- | --- | --- | --- | --- | --- | --- | --- | --- | --- |
|  |  |  |  | **669** | **729** | **730** | **731** | **732** | **733** | **734** | **735** | **736** | **737** | **738** | **739** | **740** |
| KP404149.1 | Gillingham/2012/UK | 2012 | England | - | T | F | A | L | P | T | I | E | E | E | H | L |
| KP404150.1 | Lincoln/2012/UK | 2012 | England | - | A | L | F | D | - | - | - | - | - | - | - | L |
| KX599349.1 | HUN/2012/2 | 2012 | Hungary | - | A | L | F | D | - | - | - | - | - | - | - | L |
| KX599350.1 | HUN/2012/6 | 2012 | Hungary | - | T | F | A | L | S | T | I | E | E | E | H | L |
| KX599351.1 | HUN/2012/115 | 2012 | Hungary | - | - | - | - | - | - | - | V | V | Y | E | - | L |
| KX599352.1 | HUN/2012/126 | 2012 | Hungary | - | - | - | - | - | - | - | E | T | F | D | F | L |
| KX599353.1 | HUN/2012/135 | 2012 | Hungary | - | A | L | F | D | - | - | - | - | - | - | - | L |
| MF973500.1 | CHN/2017/44 | 2017 | China | - | I | N | A | L | P | T | I | E | E | E | Q | I |
| MF973501.1 | CHN/2017/58 | 2017 | China | - | - | - | A | L | P | T | I | E | E | E | Q | I |
| MT078247.1 | MN1-USA/ORF/2017 | 2017 | USA | - | V | L | T | L | P | T | I | E | E | E | Q | L |
| MT078248.1 | MN2-USA/2017 | 2017 | USA | S | V | F | S | L | P | T | I | E | E | E | Q | L |
| MK026166.1 | DF-BC15-CAV-AUS-2017 | 2017 | Australia | S | A | L | A | L | P | T | I | E | E | E | Q | L |
| MN881999.1 | B256/China/ZJ/2019 | 2019 | China | - | I | D | V | F | P | T | I | E | E | E | Q | I |
| MN882000.1 | B290/China/ZJ/2019 | 2019 | China | - | I | D | V | F | P | T | I | E | E | E | Q | I |
| MN882001.1 | B196/China/GZ/2019 | 2019 | China | - | - | - | A | L | P | T | I | E | E | E | Q | I |
| MN882002.1 | B179/China/GZ/2019 | 2019 | China | - | - | - | A | L | P | T | I | E | E | E | Q | I |
| MN882003.1 | B239/China/ZJ/2019 | 2019 | China | - | - | - | A | L | P | T | I | E | E | E | Q | I |
| MN882004.1 | B363/China/ZJ/2019 | 2019 | China | - | V | L | T | L | P | T | I | E | E | E | Q | L |
| MN882005.1 | B234/China/AH/2019 | 2019 | China | - | V | L | T | L | P | T | I | E | E | E | Q | L |
| MN882006.1 | B157/China/HLJ/2019 | 2019 | China | - | V | L | T | L | P | T | I | E | E | E | Q | L |
| MN882007.1 | B210/China/ZJ/2018 | 2018 | China | - | A | F | A | L | P | T | I | E | E | E | Q | L |
| MN882008.1 | B091/China/GZ/2018 | 2018 | China | - | A | F | A | L | P | T | I | E | E | E | Q | L |
| MN882009.1 | B194/China/GZ/2019 | 2019 | China | - | T | F | A | L | P | T | I | E | E | E | Q | L |
| MN882010.1 | B507/China/ZJ/2019 | 2019 | China | - | A | F | A | L | P | T | I | E | E | E | Q | L |
| MT894143.1 | PVNRTVU2020/INDIA/2020 | 2020 | India | - | A | L | F | D | - | - | - | - | - | - | - | L |
| OR220030 (This study) | G21/Thailand/2021 | 2021 | Thailand | S | A | L | F | N | - | - | - | - | - | - | - | L |
| OR220029 (This study) | S76/Thailand/2021 | 2021 | Thailand | - | A | F | A | L | P | T | I | E | E | E | Q | L |
| OR220022 (This study) | V91/Vietnam/2022 | 2022 | Vietnam | - | A | F | A | L | P | T | I | E | E | E | Q | L |
| OR220023 (This study) | V194/Vietnam/2022 | 2022 | Vietnam | - | A | F | A | L | P | T | I | E | E | E | Q | L |
| OR220024 (This study) | V196/Vietnam/2022 | 2022 | Vietnam | - | A | F | A | L | P | T | I | E | E | E | Q | L |
| OR220025 (This study) | V138/Vietnam/2022 | 2022 | Vietnam | - | A | F | A | L | P | T | I | E | E | E | Q | L |
| OR220026 (This study) | V98/Vietnam/2022 | 2022 | Vietnam | - | A | F | A | L | P | T | I | E | E | E | Q | L |
| OR220027 (This study) | V107/Vietnam/2022 | 2022 | Vietnam | - | A | F | A | L | P | T | I | E | E | E | Q | L |
| OR220028 (This study) | V111/Vietnam/2022 | 2022 | Vietnam | - | A | F | A | L | P | T | I | E | E | E | Q | L |
